# Development and validation of the booklet “Home Exercise for Children and Adolescents with CharcotMarie-Tooth Disease”

**DOI:** 10.1590/1984-0462/2026/44/2026002

**Published:** 2026-10-26

**Authors:** Juliana Cardoso, Bruna Rodrigues Jordão, Wilson Marques, Cyntia Rogean de Jesus Alves de Baptista, Ana Claudia Mattiello-Sverzut

**Affiliations:** a Universidade de São Paulo, Faculdade de Medicina de Ribeirão Preto, Departamento de Ciências da Saúde, Ribeirão Preto, SP, Brazil.; b Universidade de São Paulo, Faculdade de Medicina de Ribeirão Preto, Departamento de Neurociências e Ciências do Comportamento, Ribeirão Preto, SP, Brazil.

**Keywords:** Children, Adolescents, Hereditary sensory and motor neuropathy, Health education, Crianças, Adolescentes, Neuropatia motora e sensitiva hereditária, Educação em saúde

## Abstract

**Objective:**

To develop a home-based exercise booklet for children and adolescents with CharcotMarie-Tooth disease (CMT) and to validate its content with expert judges and the target audience.

**Methods:**

This methodological study, initiated in 2019, had the booklet content developed based on the clinical experience of specialized professionals and the scientific literature. The layout was designed in accordance with recommendations for health education materials. Physiotherapists with clinical experience in child neurology and primary caregivers were included. Eleven expert judges and 15 caregivers were selected for booklet validation. Two different questionnaires were used for content validation and adherence analysis (15 and 45 days after booklet delivery). The Content Validity Index (CVI) was used to evaluate content validity among expert judges, whereas for caregivers, response frequencies were analyzed.

**Results:**

The booklet was developed in six sections, including exercises for strengthening, stretching, and balance training. Nine judges responded to the questionnaire, and all aspects assessed in the content validation achieved a minimum CVI of 0.9. Caregivers responded predominantly “agree” or “strongly agree” to all items related to the evaluation of the material. After 15 days, adherence to the proposed exercises was 53.0%, increasing to 66.0% at 45 days.

**Conclusions:**

The booklet proved to be a useful and validated tool to support the physiotherapeutic management of children and adolescents with CMT.

## RESUMO

**Objetivo:**

Desenvolver uma cartilha de exercícios domiciliares para crianças e adolescentes com doença de Charcot-Marie-Tooth (CMT) e validar seu conteúdo com juízes especialistas e público-alvo.

**Métodos:**

Este estudo metodológico, iniciado em 2019, teve o conteúdo da cartilha elaborado com base na experiência clínica de profissionais especializados e na literatura científica. O layout seguiu as recomendações para a elaboração de materiais de orientação em saúde. Fisioterapeutas com experiência clínica em neurologia infantil e cuidadores primários foram incluídos. Onze juízes especialistas e 15 cuidadores foram selecionados para a validação da cartilha. Dois diferentes questionários foram usados para a validação do conteúdo e análise de adesão à cartilha (15 e 45 dias após a entrega da cartilha). O Índice de Validade de Conteúdo (CVI) foi utilizado para avaliar a validade de conteúdo entre juízes especialistas, enquanto, para os cuidadores, foi considerada a frequência das respostas obtidas.

**Resultados:**

A cartilha foi desenvolvida em seis seções, com exercícios para fortalecimento, alongamento e treino de equilíbrio. Nove juízes responderam ao questionário proposto, e todos os aspectos abordados na validação do conteúdo alcançaram um CVI mínimo de 0,9. Os cuidadores responderam predominantemente “concordo” ou “concordo totalmente” para todos os itens referentes à avaliação do material. Após 15 dias, a taxa de adesão aos exercícios propostos foi 53,0% e, após 45 dias, de 66,0%.

**Conclusões:**

A cartilha revelou ser um instrumento válido e útil para auxiliar no tratamento fisioterapêutico de crianças e adolescentes com CMT.

## INTRODUCTION

 Charcot-Marie-Tooth disease (CMT) represents the most common group of progressive inherited peripheral neuropathies, which may follow autosomal dominant, autosomal recessive, or X-linked inheritance patterns.^1^ The disease results from mutations in genes encoding proteins involved in the structure and function of the peripheral nerve axon or the peripheral myelin sheath.^2^

 In general, clinical signs and symptoms begin within the first two decades of life and are primarily associated with somatosensory deficits and muscle weakness, more pronounced in the distal regions of the upper and lower limbs.^3^ Even in childhood, these clinical impairments lead to biomechanical gait abnormalities, foot postural deformities and balance deficits,^3-5^ which can reduce autonomy and participation in physical activities,^6^ increase the risk of falls and, consequently, result in reduced quality of life. 

 Given the absence of curative interventions for CMT, rehabilitation constitutes the main pillar of disease management, aiming to prevent the progression of clinical impairment and to promote better quality of life for patients.^7^ The international clinical practice guideline for the rehabilitation of children and adolescents with CMT recommends that treatment should include muscle strengthening exercises, muscle-joint stretching, upper limb functional training, balance training and the use of assistive technology devices.^7^ However, these recommendations are primarily directed towards healthcare professionals and are typically implemented in supervised clinical settings.^8^

 Despite the recognized importance of family-centered care in pediatric rehabilitation,^9,10^ validated educational resources for home-based use by caregivers of children and adolescents with CMT are not available. This is particularly relevant in CMT, a rare progressive disease,^11^ which requires long-term management beyond clinical settings. Providing accessible, evidence-based materials may support home-based interventions, improve adherence to therapeutic exercises, promote patient autonomy, and encourage shared responsibility between families and healthcare professionals, while reducing reliance on face-to-face physiotherapy services. 

 Therefore, the primary objective of the present study was to develop the educational booklet entitled “Home Exercise for Children and Adolescents with Charcot-Marie-Tooth Disease”. The secondary objective was to assess the content validity of the booklet with the participation of expert judges and the target audience. 

## METHOD

 In this methodological study, an educational technology, characterized as a booklet, was developed and validated. The study was approved by the Research Ethics Committee of the Hospital das Clínicas da Faculdade de Medicina de Ribeirão Preto – Universidade de São Paulo (HCFMRP–USP) (CAAE No. 59926222.5.0000.5440). All participants and their legal guardians provided written informed consent and assent, as appropriate. 

 The booklet “Home Exercise for Children and Adolescents with Charcot-Marie-Tooth Disease” was developed to guide patients and caregivers of children and adolescents with CMT undergoing treatment in outpatient rehabilitation services. Booklet development started in 2019 but, due to the COVID-19 pandemic, was only completed and validated in 2025. Five healthcare professionals participated in the development of the content: four physiotherapists and one occupational therapist. 

 The initial selection of content was based on the clinical experience of the authors from a reference rehabilitation center and on the scientific literature. Initially, the development of the strengthening exercises followed the protocol proposed by the randomized clinical trial conducted by Burns et al.,^12^ whilst the other exercises drew primarily on the clinical experience of the authors ACMS and CRJAB, who have over ten years’ experience in the rehabilitation of pediatric patients with CMT. In 2022, following the establishment of the guideline for the pediatric management of CMT,^7^ which sets out recommendations for the rehabilitation of these patients, the content of the booklet was reviewed. However, no changes were made, as the content of the booklet was already in line with the guidelines. 

 The development of the text, the creation of the figures, and the layout design followed the recommendations of Echer^13^ and Moreira et al.^14^ The text was written in a simple and descriptive manner to ensure effective communication. Rare, long, or compound words were avoided. The figures were produced by professionals based on photographs taken by the study team. All figures were designed to be clear and to accurately depict the intended behavior, particularly during performance of the exercises. In formatting the document, the Myriad Pro font (size 12) with 1.5-line spacing was selected to ensure text clarity and readability. The content was organized into sections, and the exercises were planned to be performed on up to three days per week. The design of all exercises considered a home-based setting and the use of materials commonly available at home. Prior to the final version submitted to the expert judges, two preliminary versions were produced and presented for discussion among the authors. 

 The content validation process was conducted in two parts. First, the content was evaluated by expert judges. Second, the booklet was evaluated by the target audience. Following previous studies,^10,15-17^ a convenience sample of 11 expert judges was recruited. The inclusion criteria were being a physiotherapist and having experience in child neurology. The judges were invited to participate in the study by e-mail. In the same e-mail, the booklet in PDF format and a link to access the consent form and the research questionnaire were provided. The online questionnaire consisted of 27 items divided into six categories: objectives, organization, language, appearance, motivation, and cultural adequacy.^17,18^ A description of each item is provided in the study by Franco et al.^17^ Each item was rated on a four-point scale, where

1. item not equivalent;
2. item requires major revision to assess equivalence;
3. item equivalent, requiring minor revisions; and
4. item absolutely equivalent.^17,18^

For the target audience evaluation, following previous studies,^10,17^ a convenience sample of 15 patients and their primary caregivers attending the Pediatric Charcot-Marie-Tooth Physiotherapy Outpatient Clinic at the Rehabilitation Center of the HCFMRP–USP, between October 2024 and July 2025, was recruited. 

 The inclusion criteria were a confirmed clinical diagnosis of CMT, age between five and 18 years, no history of upper or lower limb fracture or surgery within the previous six months, and absence of other conditions that could prevent the performance of the exercises proposed in the booklet. The exclusion criteria were caregiver illiteracy, withdrawal from the study, and the occurrence of an upper or lower limb injury that prevented the performance of the exercises proposed in the booklet. 

 Before receiving the booklet, participants underwent a physical assessment at the HCFMRP–USP Rehabilitation Center. Anthropometric measurements (body mass and height) were obtained, and the level of clinical impairment was assessed using the Charcot-Marie-Tooth Pediatric Scale, translated and validated into Brazilian Portuguese (CMTPedS-Br).^19,20^ The total score of this scale ranges from 0 to 44, where 0 to 14 indicate mild impairment, 15 to 29 moderate impairment, and 30 to 44 severe impairment.^11^

 At the end of the assessment, a printed booklet entitled “Home Exercise for Children and Adolescents with CharcotMarie-Tooth Disease” was provided and read in the presence of the caregiver and the child/adolescent. On the same day, a practical training session was conducted by the research team to ensure correct execution of the exercises. Patients and caregivers reported understanding the content, and any questions were clarified by the team in order to ensure the effectiveness of the training. The research team recommended that patients perform the exercises three times per week, following the order set out in the booklet. For patients who engage in physical activity or physiotherapy, the exercises were recommended to be performed on alternate days. 

 Fifteen days after delivery of the booklet and the initial training, the caregivers received two online questionnaires to evaluate the material and analyze adherence. To facilitate understanding, these questionnaires were presented to caregivers in advance on the day the booklet was delivered. For content validation of the material, the questionnaire previously used for the expert judges was applied;^17,18^ however, for caregivers, responses were presented using a Likert scale:

1. strongly disagree;
2. disagree;
3. neutral;
4. agree; and
5. strongly agree.

To analyze adherence to the exercises proposed in the booklet, a questionnaire addressing the following items was applied:^17,20^

1. Stretching exercises;
2. Strengthening exercises;
3. Balance exercises; and
4. All proposed exercises.

Each item was rated on a four-point scale, with caregivers reporting how often the exercises had been performed in the past week:^17,20^ three days per week=3; two days per week=2; one day per week=1; or no days per week=0. Finally, at the 45-day follow-up, caregivers again completed the adherence questionnaire, considering the exercises performed during the previous week. 

 For data analysis, descriptive analysis was performed using Microsoft Excel software. Frequencies were calculated for categorical variables, and minimum and maximum values were reported for numerical variables. Content validity was evaluated using the Content Validity Index (CVI).^10,17^ To calculate the CVI for each questionnaire item, responses rated as 3 or 4 by the expert judges were summed and divided by the total number of responses, according to the following formula: CVI=number of ratings of 3 or 4/total number of ratings. An acceptable level of agreement among expert judges was defined as a minimum CVI of 0.80, and preferably greater than 0.90.^21^

 To analyze content validity by caregivers, responses for each item were grouped and presented as frequency measures. For adherence analysis, an adherence score was assigned to each participant by calculating the ratio between the number of points obtained and the total number of points possible in the questionnaire. Scores greater than 0.70 were considered indicative of high adherence to treatment, whereas scores equal to or below 0.70 were classified as moderate/low adherence. These categories (high and moderate/low) were grouped and presented as frequencies. 

## RESULTS

 The booklet “Home Exercise for Children and Adolescents with Charcot-Marie-Tooth Disease” was developed with the following sections: preface; general recommendations for exercise execution; exercises for ambulatory children and for children who use wheelchairs; recommendations for daily activities; orthosis care; and adaptations of materials for activities of daily living. The exercise section included activities focused on muscle strengthening, improvement of muscle-joint mobility and postural balance. Specifically, for the ankle dorsiflexor exercise, the dosage of load and number of repetitions were left blank to be completed by the responsible healthcare professional according to each patient’s individual needs. The booklet comprised a cover page and 20 pages (Figure 1), and the digital version is available upon request by e-mail from the corresponding author. 

**Figure 1:**
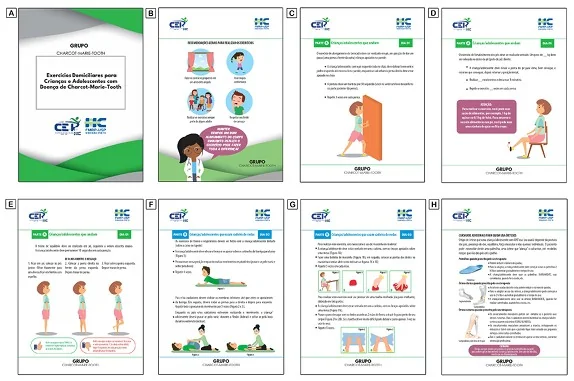
Selected pages from the booklet “Home Exercises for Children and Adolescents with Charcot-Marie-Tooth Disease”. Simplified description of the content presented on each page shown in the figure: A) booklet cover; B) general instructions for performing the exercises; C) stretching exercise for the posterior lower leg muscles; D) strengthening exercise for the ankle dorsiflexors; E) balance training exercises; F) exercises for non-ambulatory participants; G=strengthening exercises for the intrinsic hand muscles; H) guidance on orthosis use. Source: The personal archive of the authors.

 For content validation by expert judges, nine of the 11 judges who received the questionnaire completed the evaluation. The judges had completed either a specialization or another postgraduate course in child neurology, and had between eight and 22 years of clinical experience. Based on the judges’ responses, all 27 items of the booklet achieved an acceptable level of agreement, with minimum and maximum values of 0.9 and 1.0, respectively (Table 1). Therefore, no modifications were made to the version delivered to the children and adolescents and their respective caregivers. 

**Table 1:** Responses from expert judges for validation of the booklet’s content.

	Items	Item not equivalent		Item requires major revision to evaluate equivalence		Item equivalent, requiring minor revisions		Item absolutely equivalent		CVI
		n	%	n	%	n	%	n	%	
Aim	1	0	0.0	1	11.1	1	11.1	7	77.7	0.9
	2	0	0.0	1	11.1	0	0.0	8	88.8	0.9
	3	1	11.1	0	0.0	1	11.1	7	44.4	0.9
Organization	1	0	0.0	0	0.0	1	11.1	8	88.8	1.0
	2	1	11.1	0	0.0	2	22.2	6	66.6	0.9
	3	0	0.0	1	11.1	1	11.1	7	77.7	0.9
	4	0	0.0	1	11.1	0	0.0	8	88.8	0.9
	5	1	11.1	0	0.0	0	0.0	8	88.8	0.9
	6	0	0.0	1	11.1	3	33.3	8	88.8	0.9
	7	1	11.1	0	0.0	1	11.1	7	77.7	0.9
Language	1	0	0.0	1	11.1	0	0.0	7	77.7	0.9
	2	0	0.0	1	11.1	0	0.0	8	88.8	0.9
	3	0	0.0	1	11.1	0	0.0	8	88.8	0.9
	4	0	0.0	1	11.1	0	0.0	8	88.8	0.9
	5	1	11.1	0	0.0	0	0.0	8	88.8	0.9
Appearance	1	1	11.1	0	0.0	0	0.0	8	88.8	0.9
	2	0	0.0	1	11.1	0	0.0	8	88.8	0.9
	3	0	0.0	1	11.1	0	0.0	8	88.8	0.9
	4	0	0.0	1	11.1	0	0.0	8	88.8	0.9
	5	1	11.1	0	0.0	0	0.0	8	88.8	0.9
Appearance	1	1	11.1	0	0.0	0	0.0	8	88.8	0.9
	2	1	11.1	0	0.0	1	11.1	7	77.7	0.9
	3	1	11.1	0	0.0	0	0.0	8	88.8	0.9
	4	1	11.1	0	0.0	0	0.0	8	88.8	0.9
	5	1	11.1	0	0.0	0	0.0	8	88.8	0.9
Motivation	1	1	11.1	0	0.0	0	0.0	8	88.8	0.9
	2	0	0.0	0	11.1	1	11.1	8	88.8	1.0
	3	0	0.0	1	11.1	0	0.0	8	88.8	0.9
	4	1	11.1	0	0.0	0	0.0	8	88.8	0.9
	5	1	11.1	0	0.0	0	0.0	8	88.8	0.9
	6	0	0.0	1	11.1	1	11.1	7	77.7	0.9
Cultural suitability	1	0	0.0	1	11.1	0	0.0	8	88.8	0.9

CVI: content validity index; n: absolute frequency; %: relative frequency.

Source: The authors.

 A total of 15 booklets were distributed to caregivers and children/adolescents with CMT receiving outpatient care at the HCFMRP–USP Rehabilitation Center. Table 2 presents the sample characteristics, with ages ranging from five to 17 years old, and 53.3% of participants were male. Among the patients, 12 were undergoing physiotherapy, and ten participated in regular physical activity. The total CMTPedS-Br scores ranged from 4 to 33 points (Table 2). Among the caregivers, 93.0% were mothers, 20.0% had not completed primary education, 13.3% had completed primary education, 33.3% had completed secondary education, and 33.3% had completed higher education. 

**Table 2:** Characterization of the sample.

Identification	Sex	Age (years)	Weight (kg)	Height(cm)	CMTPedS-Br total score	CMT type
1	M	9	36.2	141.0	22	1A
2	F	9	35.8	135.5	10	1A
3	M	14	75.3	172.0	19	1A
4	M	10	31.3	133.5	17	1A
5	F	13	56.7	162.0	32	2A
6	F	17	56.5	162.0	14	1A
7	F	5	16.3	112.0	9	2A
8	F	5	16.2	112.0	32	Genetically undiagnosed
9	M	15	67.9	177.0	27	2A
10	F	13	50.0	154.0	17	1A
11	M	17	66.2	186.0	17	1A
12	F	9	27.3	141.5	33	1A
13	M	12	76.7	152.5	18	1A
14	M	14	52.7	166.0	20	1A
15	M	8	57.9	142.0	4	1A

CMTPedS-Br: Charcot-Marie-Tooth Pediatric Scale (Brazilian version); F: female, M: Male.

Source: The authors.

 For content validation by caregivers, the responses “agree” and “strongly agree” were the most frequent across items (Table 3). 

**Table 3:** Frequency table of caregiver responses for the evaluation of the material.

	Items	Strongly disagree		Disagree		Neutral		Agree		Strongly agree	
		n	%	n	%	n	%	n	%	n	%
Aim	1	0	0.0	0	0.0	1	6.6	6	40.0	8	53.3
	2	0	0.0	0	0.0	1	6.6	6	40.0	8	53.3
	3	0	0.0	0	0.0	0	0.0	7	46.6	8	53.3
Organization	1	0	0.0	0	0.0	1	6.6	9	60.0	5	38.4
	2	0	0.0	0	0.0	0	0.0	9	60.0	6	40.0
	3	0	0.0	0	0.0	0	0.0	8	53.3	7	46.6
	4	0	0.0	0	0.0	1	6.6	8	53.3	6	40.0
	5	0	0.0	0	0.0	3	20.0	6	40.0	6	40.0
	6	0	0.0	0	0.0	3	20.0	6	40.0	6	40.0
	7	0	0.0	0	0.0	0	0.0	7	46.6	8	53.3
Language	1	0	0.0	0	0.0	1	6.6	7	46.6	7	46.6
	2	0	0.0	0	0.0	2	13.3	6	40.0	7	46.6
	3	0	0.0	0	0.0	1	6.6	6	40.0	8	53.3
	4	0	0.0	0	0.0	0	0.0	7	46.6	8	53.3
	5	0	0.0	0	0.0	0	0.0	5	33.3	10	66.6
Appearance	1	0	0.0	0	0.0	0	0.0	7	46.6	8	53.3
	2	0	0.0	0	0.0	0	0.0	9	60.0	6	40.0
	3	0	0.0	0	0.0	1	6.6	8	53.3	6	40.0
	4	0	0.0	0	0.0	1	6.6	7	46.6	7	46.6
	5	0	0.0	1	6.6	8	53.3	3	20.0	3	20.0
Motivation	1	0	0.0	0	0.0	1	6.6	8	53.3	6	40.0
	2	0	0.0	1	6.6	4	26.6	8	53.3	2	13.3
	3	0	0.0	0	0.0	0	0.0	9	60.0	6	40.0
	4	0	0.0	0	0.0	0	0.0	7	46.6	8	53.3
	5	0	0.0	0	0.0	1	6.6	7	46.6	7	46.6
	6	0	0.0	1	6.6	4	26.6	6	40.0	4	26.6
Cultural suitability	1	0	0.0	0	0.0	1	6.6	7	46.6	7	46.6

n: absolute frequency; %: relative frequency.

Source: The authors.

 Regarding adherence, eight participants (53.0%) demonstrated “high” adherence, with scores >0.7, 15 days after booklet delivery. At the 45-day follow-up, this proportion increased to ten participants (66.0%) (Figure 2). 

**Figure 2:**
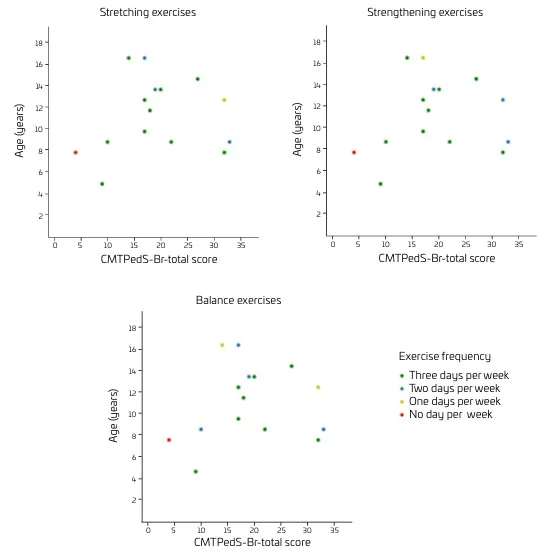
Exercise frequency reported by children and adolescents with Charcot-Marie-Tooth disease at the 45-day follow-up, according to age and clinical impairment. CMTPedS-Br: Charcot-Marie-Tooth Pediatric Scale (Brazilian version). Source: The authors.

## DISCUSSION

 This study developed and validated a home-based exercise booklet to support the rehabilitation of children and adolescents with CMT. The availability of this material has the potential not only to enhance rehabilitation outcomes through a family-centered care approach, but also to improve the efficiency of clinical services and extend access to individuals who may not have access to specialized CMT rehabilitation. The use of this booklet may help bridge the gap between scientific evidence and everyday practice by enabling patients and their families to implement evidence-based exercises independently. 

 Our home-based exercise booklet is the first material designed for children and adolescents with CMT to be developed following a standardized methodology. Although educational materials for the general CMT population can be found online,^22-24^ these are part of the grey literature and have not undergone formal validation processes. While such resources may support rehabilitation to some extent, the lack of standardization and scientific validation may limit their reliability and consistency. In this context, the development and validation of structured educational materials are essential to ensure the accuracy and safety of the information provided. 

 In this study, the booklet demonstrated adequate content validity according to expert judges and the target audience. Although 11 expert judges were invited, only nine completed the content validation process, which may have slightly reduced the diversity of perspectives. Nevertheless, the participation of experts and target audience allows for a more comprehensive evaluation of the material, ensuring clinical and practical relevance.^25^ The comparison of our findings with the literature is limited; however, studies that have developed home-based exercise booklets using a similar methodological process for children and adolescents with chronic conditions have also reported adequate content validity.^10,17^

 Regarding the content validity obtained through the target audience, it is important to note that our sample included only ambulatory patients. Although no wheelchair users participated in the study, this likely had a limited impact on the overall content of the booklet, as home-based exercises for non-ambulatory individuals are generally less disease-specific and primarily focused on maintaining mobility, posture, and upper-limb function. Furthermore, the exercises included in our booklet for non-ambulatory patients had previously been incorporated into educational materials developed by our research team, for which content validity had already been evaluated by the target audience.^10,17^

 For adherence, 53.0% of participants demonstrated “high” adherence 15 days after booklet delivery, increasing to 66.0% at the 45-day follow-up. These findings differ from those reported in the literature for pediatric patients with Duchenne muscular dystrophy (DMD) and spina bifida, in which adherence was low after 15 days of booklet use and decreased even further after 45 days.^10,17^ One possible explanation for this difference is the more severe clinical impairment observed in the samples of Santos et al.^10^ and Franco et al.,^17^ which may hinder engagement with prescribed exercises. 

 Despite the increase in adherence at the 45-day follow-up, full adherence across the sample was not achieved. Our sample showed a heterogeneous distribution of adherence when considering both clinical severity and age, and caregivers also presented variability in educational level. The literature shows that educational materials targeted at pediatric populations, when developed in a clear and accessible manner, can promote knowledge acquisition and active participation in care.^26^ However, the implementation of home-based exercise programs remains challenging, even in more prevalent conditions such as cerebral palsy.^9^ Sociodemographic factors, levels of motivation, social support, and environmental conditions have been identified as important determinants of adherence to home-based rehabilitation practices.^27^ For pediatric neuromuscular diseases, further research with larger sample sizes is needed to better understand these factors and optimize adherence strategies. 

 Finally, our booklet was intentionally developed in a printed format to provide a simple and accessible resource for families and clinical services. A PDF version is also available to facilitate dissemination and its use in research and clinical practice. Future studies may explore potential adaptations to digital platforms. These different formats could incorporate features such as automatic reminders, instructional videos, activity logs, and real-time feedback, potentially reducing barriers such as forgetfulness, uncertainty during exercise execution, and reduced motivation, while also facilitating adherence and enabling remote monitoring by healthcare professionals. 

 As limitations, the scientific evidence used to develop the booklet was identified through a search of a single database (United States National Library of Medicine — PubMed), which may have limited the inclusion of studies. The number of expert judges was relatively small, although it is consistent with previous studies.^10,15-17^ Additionally, the target audience sample was small and heterogeneous, which reflects the rarity of CMT. No wheelchair users were included; therefore, the content validity of the exercises designed for this subgroup was not evaluated by the target audience. 

 In conclusion, the exercise booklet for children and adolescents with CMT demonstrated satisfactory content validity, good acceptability among caregivers, and promising adherence over time. The booklet represents a feasible resource to support homebased physiotherapeutic management in pediatric CMT and highlights the importance of structured educational materials as part of rehabilitation strategies for rare neuromuscular conditions. 

## Data Availability

The database that originated the article is available with the corresponding author.
